# HdeB chaperone activity is coupled to its intrinsic dynamic properties

**DOI:** 10.1038/srep16856

**Published:** 2015-11-23

**Authors:** Jienv Ding, Chengfeng Yang, Xiaogang Niu, Yunfei Hu, Changwen Jin

**Affiliations:** 1 College of Life Sciences, Peking University, Beijing 100871, China; 2 College of Chemistry and Molecular Engineering, Peking University, Beijing 100871, China; 3 Beijing Nuclear Magnetic Resonance Center, Peking University, Beijing 100871, China; 4 Beijing National Laboratory for Molecular Sciences, Peking University, Beijing 100871, China

## Abstract

Enteric bacteria encounter extreme acidity when passing through hosts’ stomach. Since the bacterial periplasmic space quickly equilibrates with outer environment, an efficient acid resistance mechanism is essential in preventing irreversible protein denaturation/aggregation and maintaining bacteria viability. HdeB, along with its homolog HdeA, was identified as a periplasmic acid-resistant chaperone. Both proteins exist as homodimers and share similar monomeric structures under neutral pH, while showing different dimeric packing interfaces. Previous investigations show that HdeA functions through an acid-induced dimer-to-monomer transition and partial unfolding at low pH (pH 2–3), resulting in exposure of hydrophobic surfaces that bind substrate proteins. In contrast, HdeB appears to have a much higher optimal activation pH (pH 4–5), under which condition the protein maintains a well-folded dimer and the mechanism for its chaperone activity remains elusive. Herein, we present an NMR study of HdeB to investigate its dynamic properties. Our results reveal that HdeB undergoes significant micro- to milli-second timescale conformational exchanges at neutral to near-neutral pH, under the later condition it exhibits optimal activity. The current study indicates that HdeB activation is coupled to its intrinsic dynamics instead of structural changes, and therefore its functional mechanism is apparently different from HdeA.

Enteric Gram-negative bacteria utilize an intricate acid resistance system for self-protection when passing through the highly acidic mammalian gastric environment (pH 1–3)^1^,^2^,^3^. Due to the permeability of bacterial outer membrane, periplasmic proteins are quickly exposed to low pH during acid stress and thus prone to acid-induced denaturation and/or aggregation. To maintain viability, enteric bacteria such as *Escherichia coli* and *Helicobacter pylori* have evolved a specialized periplasmic acid resistance system involving several identified chaperones, including DegP, SurA and two essential proteins HdeA and HdeB^2^,^3^,^4^,^5^,^6^. Among these, the HdeA/B proteins are acid-induced chaperones that protect substrate proteins from aggregation and subsequently help substrate refolding upon pH neutralization^4^,^5^.

The functional mechanism of HdeA has been subjected to extensive biochemical, structural and computer simulation studies in recent years^3^,^4^,^7^,^8^,^9^,^10^,^11^,^12^,^13^,^14^,^15^,^16^. It is classified as a ‘conditionally-disordered’ protein, adopting a well-folded dimer structure under neutral conditions (pH > 3.5), which is functionally inactivated, and undergoing an acid-induced partial unfolding event accompanied by dimer dissociation which leads to chaperone activation^12^,^13^,^14^,^15^,^16^. The protonation of a number of negatively charged residues was identified to contribute to the dimer destabilization and protein unfolding^12^,^13^,^14^, and significantly increased hydrophobic surface exposure observed at low pH conditions was believed to play a main role in binding substrate proteins and preventing them from aggregation^7^,^10^. Upon pH neutralization, HdeA is suggested to facilitate substrate refolding via a ‘slow-release’ mechanism, while HdeA itself refolds into the inactive dimer^11^.

Though sharing 35% sequence similarity with HdeA, HdeB is less well characterized and its functional mechanism remains unclear. Both proteins were demonstrated to undergo concomitant monomerization and partial unfolding at low pH (<3), but show considerable differences in their chaperone activities^5^. They are shown to help reduce the aggregation size and hydrophobic surface area of substrate proteins^8^, but the exposed hydrophobic surface of HdeB at low pH is much less compared to HdeA^5^. On the other hand, the activity of HdeA in preventing substrate aggregation is optimal at pH 2^5^, whereas HdeB is shown to have optimal chaperone activity at near-neutral pH range (pH 4–5), under which condition HdeA is completely inactive^17^. These evidences together suggest that the functional mechanism of HdeB in acid stress is significantly different from HdeA.

Crystal structures of HdeA and HdeB at near-neutral pH have been determined^4^,^18^,^19^. Both proteins form homo-dimers, showing highly similar monomer structures but different dimer interfaces. NMR titration and computational investigations on HdeA revealed an acid-induced structural loosening correlated with the proposed mechanism that dimer dissociation and partial unfolding at low pH activates the chaperone function^12^,^13^,^14^,^15^,^16^. In contrast, HdeB remains a well-folded dimer at pH 4^19^, and a preliminary NMR pH-titration data do not suggest large structural changes of HdeB during the decrease of pH from neutral to near-neutral range, the later shown to be the active pH for HdeB function^17^. Analytic ultracentrifugation results suggest that HdeB is highly dynamic and may undergo structural rearrangements without dimer dissociation during the pH transition from neutral to near-neutral range^17^. However, little is known about detailed conformational and/or dynamic changes during HdeB activation, and current available structural data cannot readily explain the distinct chaperone activities of the HdeA/B homologs. To understand the molecular mechanisms of HdeA/B functions, a key question awaits to be answered: Why is HdeA activation coupled to partial unfolding and dimer dissociation at low pH, whereas HdeB is active in its dimeric form at near-neutral pH without undergoing significant structural changes?

Protein functions are closely correlated to both their structures and dynamic properties^20^,^21^,^22^,^23^,^24^. In particular, the recognition and interaction between chaperon proteins and substrates are highly dynamic, and fluctuations of both structures and/or dynamics are coupled to chaperon activation^25^. During HdeA activation, partial unfolding and dimer-to-monomer dissociation result in changes of both protein structure and dynamics. In the case of HdeB, the dimeric structure keeps almost unchanged from inactive to active pH. Consequently, its activation most probably associates with the changes of dynamics. Herein, we present a systematic solution NMR study on the structure and dynamics of *E. coli* HdeB during its activation. Our results show that HdeB maintains a folded dimer structure in the neutral to near-neutral pH range (pH 7–3.5), whereas significant pH-dependent conformational exchanges are observed in the α2-α3 loop at the dimer interface. The highly dynamic feature of HdeB at near-neutral pH is in contrast to the much higher structural rigidity of HdeA. Based on our current results in combination with previous studies, a ‘spring clamp’ mechanism for HdeB chaperone activity is proposed.

## Results

### pH titration of HdeB monitored by NMR

**Figure 1:**
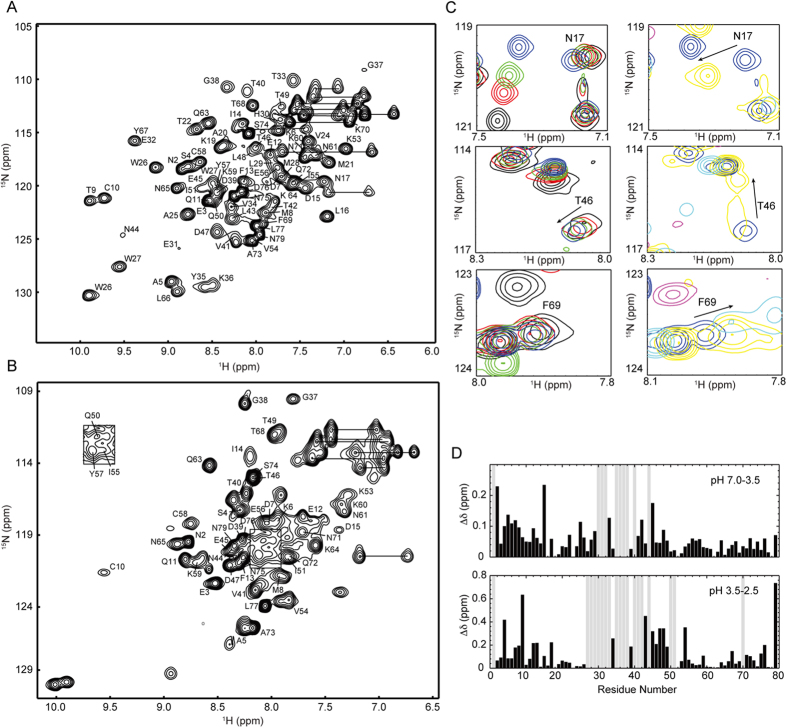
pH-dependent conformational changes of *E. coli* HdeB characterized by NMR. (**A**–**B**) 2D ^1^H-^15^N HSQC spectra of *E. coli* HdeB at pH 4.5 (**A**) and 1.5 (**B**) with backbone resonance assignments labeled. (**C**) Overlay of ^1^H-^15^N HSQC spectra of representative residues over the pH ranges of 7.0–3.5 (left panel) and 3.5–1.5 (right panel). The spectra are colored as: pH 7.0, black; pH 4.5, red; pH 4.0, green; pH 3.5, blue; pH 2.8, yellow; pH 2.0, magenta; pH 1.5, cyan. (**D**) Composite chemical shift changes between pH 7.0–3.5 and 3.5–2.5. The composite chemical shift changes were calculated using the empirical equation 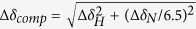. Grey bars indicate residues that are missing in one (or both) of the spectra.

It has been demonstrated that, similar to HdeA, HdeB is dominated by the dimeric conformation at neutral pH and dissociates into monomers at pH 2–3, accompanied by simultaneous partial unfolding^19^. A preliminary NMR pH titration study covering the pH range of 6.8 to 2.2 has been reported recently, which reveals significant chemical shift changes between pH 5.6 and 2.8 and a drastic spectral change (disappearance of a large number of peaks) at pH 2.2^17^. To obtain residue-specific information of HdeB structural changes during acid stress, we performed the pH titrations monitored by 2D NMR covering a pH range of 7.0–1.5 (Supplemental Fig. S1) and obtained the backbone resonance assignments at pH 4.5 (Fig. 1A). With the help of a few triple resonance experiments collected at pH 2.8, the resonances in the 2D spectra can be traced in the pH range of 7.0 to 2.8. For pH 1.5, the backbone assignments were independently obtained for 46 out of 79 residues (Fig. 1B).

During pH titration, we observed two stages of conformational changes. The pH around 3.5 appears to be a transition point for the acid-induced structural change of HdeB, since the trends of changes for the amide signals are different in the 7.0–3.5 and 3.5–1.5 ranges for many residues (Fig. 1C). When pH decreases from 7.0 to 3.5, the 2D ^1^H-^15^N HSQC spectra show gradual but moderate changes. Apart from slight chemical shift changes for residues in the N- and C-termini (Fig. 1D), a number of amide signals that are missing at pH 7.0 become observable at pH ~4.5–3.5, including Glu31, Lys36, Gly37, Asp39, Asn44, Leu48, Tyr57, Lys59 and Gln72, most of which locate on the α2-α3 loop that forms part of the dimeric interface. When pH further decreases, drastic changes were observed throughout the protein sequence which most probably correlate to a global unfolding event. Many residues show significant chemical shift perturbations from pH 3.5 to 2.5 (Fig. 1D), and a subset of residues show two or more sets of peaks in the pH range of 3.2 to 2.0, suggesting the co-existence of multiple conformations (Fig. S1). At pH 2.0–1.5, the protein reaches a generally unfolded state, with limited resonance distribution and strong signals clustering in the center of the spectra (Fig. 1B). In addition, a reverse pH titration from pH 1.5 to 7.0 demonstrates that the pH-induced conformational changes of HdeB are reversible.

### Solution Structure of HdeB Dimer at pH 4.5

**Figure 2:**
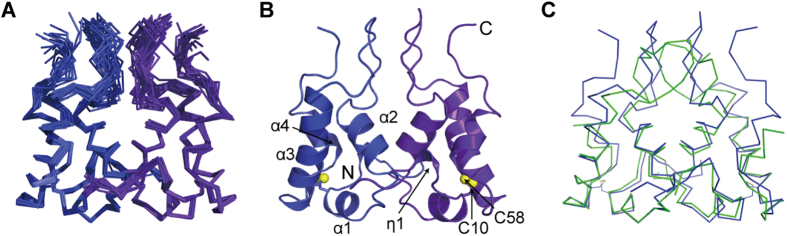
Solution structures of *E. coli* HdeB. (**A**,**B**) 20 lowest-energy structure ensemble (**A**) and ribbon diagram (**B**) of *E. coli* HdeB dimeric structure at pH 4.5. The secondary structures are labeled and the Cys10-Cys58 disulfide bonds are shown in (**B**). (**C**) Superimposition of the C^α^ trace of solution structure (blue) with crystal structure (green) (PDB ID 2XUV).

The X-ray structure of *E. coli* HdeB dimer has been solved at pH 4.5, showing an essentially similar monomeric structure with HdeA but different dimer packing interfaces^17^. To confirm that the difference is indeed not a result of crystal packing, we resolved to determine the solution NMR structure of HdeB dimer at pH 4.5, which is identical with the crystallization pH and also shows the most complete backbone resonances. The ensemble and ribbon diagram of HdeB solution structure are shown in Fig. 2A,B and the structural statistics are summarized in Table 1.

As shown in Fig. 2C, the solution structure of HdeB is generally similar to the crystal structure. Each monomer contains four α-helices (α1: Cys10-Phe13; α2: Met21-Leu29; α3: Glu45-Asn61; α4: Leu66-Asn71) and a disulfide bridge between Cys10-Cys58. In addition, residues Ala5-Asp7 in the N-terminal loop form a small one-turn helical conformation (η1). The residues are numbered according to the sequence of the mature protein, with the signal peptide excluded. The dimer interface is formed by the near-perpendicular packing of helix α2 and the following extended loop (His30-Asn44) connecting α2 and α3. The major differences between solution and crystal structures reside in the α2-α3 loop and the C-terminus, both of which are highly flexible regions. The α2-α3 loop shows an intertwined conformation in the X-ray structure, whereas it is more extended in the NMR structure. The C-terminus is invisible in the X-ray structure, whereas it is observed to pack onto the α2-α3 loop in the NMR structure, evidenced by a network of NOE contacts between residues Asn75-Pro78 on the C-terminal tail and residues Tyr35, Lys36 and Asn44 on the α2-α3 loop.

### Backbone Dynamics of HdeB at different pH

**Figure 3:**
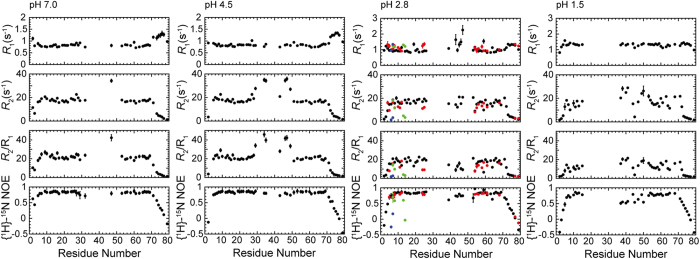
pH-dependent backbone relaxation parameters of *E. coli* HdeB. The backbone ^15^N relaxation parameters *R*_*1*_, *R*_*2*_, *R*_*2*_*/R*_*1*_ and {^1^H}-^15^N NOE values of HdeB at pH 7.0, 4.5, 2.8, and 1.5 are shown from left to right. For residues showing multiple sets of peaks at pH 2.8, the relaxation parameters of the minor conformations are colored.

To provide more detailed information on the dynamic properties of HdeB, we performed backbone ^15^N relaxation measurements of HdeB at nine different pH conditions ranging from 7.0 to 1.5, including the longitudinal relaxation rates (*R*_*1*_), transverse relaxation rates (*R*_*2*_), and steady-state heteronuclear {^1^H}-^15^N NOE values. A representative comparison of the relaxation parameters as well as the *R*_*2*_*/R*_*1*_ ratios at pH 7.0, 4.5, 2.8 and 1.5 is shown in Fig. 3, and a more extensive comparison is presented in Supplemental Fig. S2.

The ^15^N backbone relaxation data indicate that HdeB adopts a rigid structure in the dimeric form in the pH range of 7 to 4, as reflected by the mostly unchanged average *R*_*2*_*/R*_*1*_ ratios (reflecting the overall tumbling time of the molecule and thus the apparent molecular weight^26^,^27^,^28^,^29^) and the high {^1^H}-^15^N NOE values (reflecting internal flexibility on the ps-ns timescales) for most of the residues in the core secondary structures. Though a number of residues show multiple sets of cross-peaks at pH 3.2 and 2.8, the *R*_*2*_*/R*_*1*_ ratios for the main peak set remains similar to the observed values above pH 4, indicating that the major remains a folded dimer under the experimental condition (protein concentrations ~1 mM). Most of the minor peak sets show significantly lower *R*_*2*_*/R*_*1*_ ratios and {^1^H}-^15^N NOE values, which strongly suggests partial unfolding and/or dissociation thus increased local flexibility. At lower pH range of 2.5 to 1.5, the average *R*_*2*_*/R*_*1*_ ratio gradually decreases, reflecting further unfolding of the protein, which is probably accompanied by the dimer-to-monomer transition suggested in previous studies^5^,^19^. Under extremely acidic condition (pH = 1.5), the spectrum shows one set of peaks representing a stable partially unfolded state. The relaxation data supports a scenario that HdeB maintains a well-folded dimer structure under neutral and near-neutral pH conditions, and starts to undergo partial unfolding at a pH value of approximately 3^5^,^17^,^19^.

### Model-free analysis of HdeB at neutral to near-neutral pH

**Figure 4:**
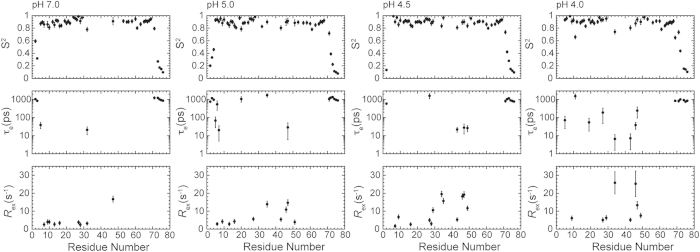
Model-free ananysis of *E. coli* HdeB backbone dynamics at neutral and near-neutral pH. The motional parameters of HdeB at pH 7.0, 5.0, 4.5 and 4.0 extracted by model-free formalism are shown from left to right. The parameters include the squared order parameter *S*^*2*^, the correlation time (*τ*_*e*_) of internal motion, and the chemical/conformational exchange contribution *R*_*ex*_.

Since the dimeric structure of HdeB remains almost unchanged in the neutral to near-neutral pH range, we used model-free formalism to extract the internal motional parameters from the ^15^N relaxation data at pH 7.0, 5.0, 4.5 and 4.0. The results are summarized in Fig. 4.

During the data analysis, 44, 49, 49 and 45 out of 79 residues were used for pH 7.0, 5.0, 4.5 and 4.0, respectively. The unanalyzed residues include the prolines, the ones unassigned, the peaks overlapped or those too weak to be accurately analyzed. For all pH conditions, the rotational diffusion tensors describing the overall tumbling of the protein molecule were best described by the oblate axially symmetric model, with the rotational correlation time of *τ*_*m*_ = 10.8 ± 0.05, 10.5 ± 0.06, 10.7 ± 0.05, 10.9 ± 0.06 ns, and diffusion anisotropy of *D*_*//*_*/D*_*⊥*_ = 0.89 ± 0.04, 0.86 ± 0.04, 0.87 ± 0.03, 0.83 ± 0.04 for pH 7.0, 5.0, 4.5 and 4.0, respectively. These results are in good correlation with the HdeB dimer structure that shows an inertia tensor of *Ix:Iy:Iz*= 1:0.74:0.61, and support that HdeB exists as a dimer in the above pH range.

At neutral pH (pH = 7.0), a total of 25 residues are assigned to model M1, with an average *S*^*2*^ = 0.91 ± 0.04. Two residues (Ala5 and Asn71) are assigned to model M2, with an average *S*^*2*^ = 0.82 ± 0.01 and internal motions on the ps-ns time scales. Eight residues (Asp7, Thr9, Cys10, Phe13, Leu16, Trp27, Met28, Asp47) are assigned to model M3, with an average *S*^*2*^ = 0.88 ± 0.06 and conformational exchanges (*R*_*ex*_) on the μs-ms time scales. One residue (Glu32) is assigned to model M4 with an *S*^*2*^ = 0.78 ± 0.03 and structural flexibility on both ps-ns and μs-ms time scales. Moreover, six residues mostly locating in the N- and C-termini (Asn2, Glu3, Ala73, Ser74, Asn75, Asp76) are assigned to model M5 with an average *S*^*2*^ = 0.27 ± 0.18. Notably, a large number of residues locating in the α2-α3 loop at the dimer interface are not included in the model-free analysis due to missing signals, indicating intermediate conformational exchanges on μs-ms time scales.

As the pH lowers to near-neutral range (pH 4.0–5.0), more residues at the dimer interface become detectable. Model-free analyses of the ^15^N relaxation data acquired at pH 5.0, 4.5 and 4.0 show generally similar results. At pH 4.5 when the protein NMR spectrum shows the most complete backbone resonances, a total of 28 residues are assigned to model M1, with an average *S*^*2*^ = 0.90 ± 0.04. Eight residues (Asp7, Thr9, Leu16, Met28, Leu29, Val34, Tyr35, Thr46) are assigned to model M3, with an average *S*^*2*^ = 0.89 ± 0.04 and conformational exchanges (*R*_*ex*_) on the μs-ms time scales. Four residues (Trp27, Leu43, Asp47, Thr49) are assigned to model M4 with an average *S*^*2*^ = 0.87 ± 0.06 and structural flexibility on both ps-ns and μs-ms time scales. Finally, seven residues in the N- and C-termini (Asn2, Asn71, Gln72, Ala73, Ser74, Asn75, Asp76) are assigned to model M5 with an average *S*^*2*^ = 0.28 ± 0.23.

Comparison of the model-free parameters extracted at different pH suggests an overall similar dynamic behavior of HdeB in the neutral to near-neutral pH range. Apart from the highly flexible N- and C-termini, the α2-α3 loop also shows fast timescale (ps-ns) conformational flexibility as reflected by the relatively low *S*^*2*^ values. In addition, the dimer-packing interface including the helix α2 and the extended α2-α3 loop exhibits significant μs-ms timescale conformational exchanges over the pH conditions examined, which is in contrast to the overall rigidity observed for the homologous yet functionally divergent chaperone HdeA^14^.

### Conformational exchanges of HdeB at active pH by relaxation dispersion experiments

The above results indicate that the α2-α3 loop exhibits significant conformational exchanges in the μs-ms timescales at neutral to near-neutral pH conditions, particularly at pH 4.5 and 4.0, which are the functionally active pH of HdeB in cell^17^. To further characterize the μs-ms timescale dynamics, we performed the backbone ^15^N CPMG relaxation dispersion experiments^30^,^31^ at pH 6.0, 4.5 and 4.0 to extract more detailed information. Possible contributions of dimer-monomer equilibrium were excluded by measuring CPMG relaxation dispersion experiments using samples with HdeB concentrations ranging from 0.5 to 2.0 mM, which showed essentially similar data. Final analyses were performed using data collected with 1.0 mM sample on both 600- and 800-MHz NMR spectrometers. All data were analyzed using a simplified two-state exchange model^32^,^33^,^34^, and the following parameters concerning the exchange between the conformations A and B could be obtained: the exchange rate k_ex_ = k_AB_+k_BA_, the populations p_A_ and p_B_, and the chemical shift difference Δω between A and B (Supplemental Fig. S3 and Table S1).

**Figure 5:**
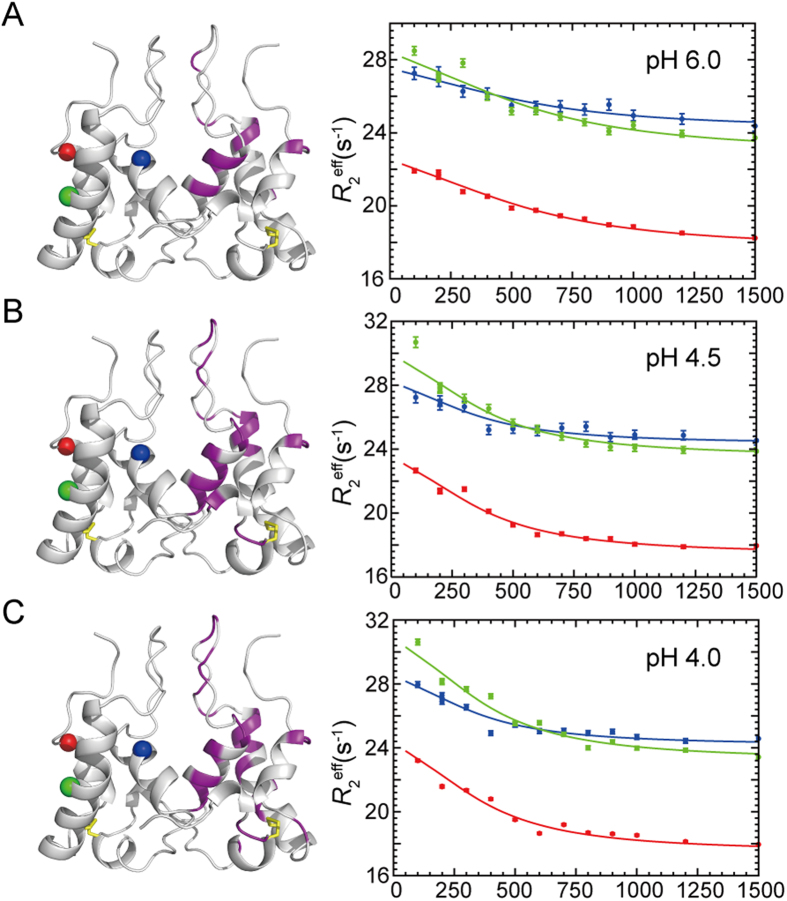
Micro- to milli-second timescale conformational exchanges of *E. coli* HdeB at neutral and near-neutral pH. Residues showing conformational exchanges at pH 6.0 (**A**), 4.5 (**B**) and 4.0 (**C**) were identified by backbone ^15^N CPMG relaxation dispersion experiments and mapped onto the structure (magenta). The relaxation dispersion profiles of representative residues Trp26 (blue), Lys53 (green) and Asn71 (red) are shown in the right panel. The locations of these three residues in the structure are shown in spheres and colored correspondingly.

As shown in Fig. 5, at pH 6.0, 4.5 and 4.0, most residues showing conformational exchanges are clustered near the dimeric interface, including the N-terminal loop, the dimer forming helix α2, the α2-α3 loop, and the tips of contacting helices α3 and α4. These residues generally show similar k_ex_ and p_A_p_B_ values and can be considered undergoing a concerted conformational fluctuation event. Global fitting using the data from all these clustered residues gives an exchange rate of k_ex_ = 1822 ± 62 s^−1^ for pH 6.0, and a decreased exchange rate of k_ex_ = 1095 ± 37 s^−1^ and 1095 ± 44 s^−1^ for pH 4.5 and 4.0, respectively. On the other hand, the populations of major and minor conformations are essentially the same for these pH conditions, with p_A_p_B_ = 0.026 ± 0.002 for pH 6.0, and p_A_p_B_ = 0.024 ± 0.001 and 0.028 ± 0.002 for pH 4.5 and 4.0, respectively. Both correspond to a 97.1–97.5% major population and a 2.5–2.9% minor population based on the two-state exchange model. The μs-ms conformational fluctuation is highly similar at pH 4.5 and 4.0 concerning both the number and distribution of residues. However, more residues show conformational exchanges at pH 4.5/4.0 compared to 6.0. The additional residues locate in the N-terminus (Ala5, Met8, Thr9) and the N-terminal half of helix α2 (Met21, Thr22, Ala25), suggesting conformational fluctuations occurring on a larger scale as the pH lowers. On the other hand, a large number of residues in the α2-α3 loop display very weak signals at pH 6.0, rendering their relaxation dispersion data unavailable, therefore conformational exchanges on μs-ms timescales cannot be excluded for these residues.

## Discussion

The HdeA and HdeB proteins have been identified as molecular chaperones that function during acid stress^4^,^5^. A recent report demonstrates that HdeB has optimal chaperone activity at pH 4–5, which is much more neutral compared to the activation pH of HdeA^17^. Up to date, the proposed HdeA chaperone mechanism involves an acid-induced partial unfolding event coupled to drastic structural changes^7^,^9^,^10^,^13^,^14^,^15^,^16^, which results in the exposure of buried hydrophobic surfaces for binding denatured substrate proteins. In contrast, HdeB apparently adopts a distinct mechanism since it retains a well-folded dimer structure at its functional active pH (4–5). The molecular mechanism of HdeB is largely unknown, and it remains enigmatic how a well-folded HdeB dimer carries out the chaperone function.

Experimental evidence from analytic ultracentrifugation suggests a mechanism involving substantial structural rearrangement of HdeB during the transition from neutral pH to the active pH of 4–5 without disrupting the dimeric packing^17^. In particular, the sedimentation coefficients were observed to be 1.5 S and 1.2 S at pH 7 and pH 2, corresponding to predominantly dimer and monomer conformations, respectively. Intriguingly, the sedimentation coefficient increased to 1.9 S at pH 4–5, indicating that the HdeB protein may occupy a larger volume compared to a dimer. The reason for this observation is yet unclear. One possibility is that HdeB undergoes significant structural rearrangement that changes the global shape of the dimer. Alternatively, it is also possible that the motional properties of HdeB change from pH 7 to pH 4–5, which affects the overall dynamic behavior and sedimentation rate.

The NMR-monitored pH titration experiments presented here and in the previous report^17^ show no indication of drastic structural changes from pH 7 to pH 5–4. Furthermore, model-free analyses of the backbone ^15^N relaxation data reveal similar overall tumbling correlation time (*τ*_*m*_) and diffusion anisotropy (*D*_*//*_*/D*_*⊥*_) from pH 7 to pH 4, supporting the scenario that the HdeB dimer does not experience significant overall structural changes during activation. Comparison of the HdeB NMR structure with crystal structure (both determined at pH 4.5) indicates that the α2-α3 loop adopts a more extended conformation in solution. On the other hand, our NMR data clearly show μs-ms timescale conformational exchanges at and near the dimeric interface, particularly in the extended α2-α3 loop. These observations together suggest that the α2-α3 loop may sample a relatively large conformational space. In addition, relaxation dispersion data reveals that the rate of the conformational exchanges apparently varies with pH, strongly suggesting that pH-dependent changes of protein dynamics may be a main role player in the activation of HdeB chaperone function. It is also highly probable that the magnitude of motion in the α2-α3 loop varies with pH, which could facilitate binding with substrate proteins. This, however, remains to be further investigated, preferably with the help of computational simulation methods.

Further inspection on the amino acid composition of the α2-α3 loop (comprising of residues H_30_EETVYKGGDTVTLNETDL_48_) shows that the segment contains a large number of charged and hydrophobic residues. A total of six charged residues are present, among which five are negative charged. Five hydrophobic residues (two valines, two leucines and one tyrosine) are evenly distributed in the segment. In addition, the methyl groups of four threonines, as well as the long side chain of the lysine residue, may also contribute to the local hydrophobic environment of this region. Considering the fact that the HdeB homo-dimer is well maintained at pH 4–5, the helix α2 at the center of the dimeric interface may not undergo very large structural movement and cannot be fully exposed. Otherwise, significant changes of the overall tumbling correlation time (*τ*_*m*_) and diffusion anisotropy (*D*_*//*_*/D*_*⊥*_) should be observed. The conformational exchanges in helix α2 could be due to the relatively large motion of the adjacent α2-α3 loop, which is the most accessible region of the HdeB dimer and can provide both hydrophobic and electrostatic interactions for binding acid-denatured substrate proteins. Moreover, the close proximity of two α2-α3 loops in the HdeB dimer may have a chelate effect, increasing the local hydrophobic area to facilitate its chaperone activity.

**Figure 6:**
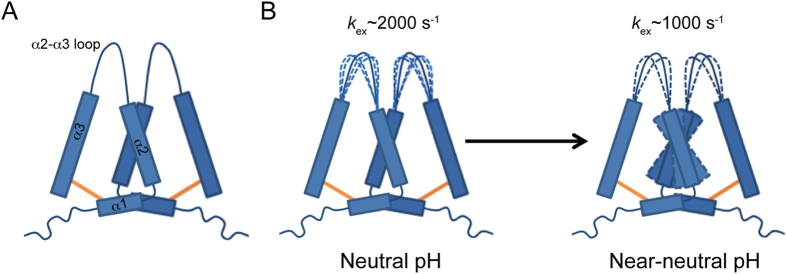
A model for *E. coli* HdeB chaperone mechanism. (**A**) A schematic presentation of the HdeB dimeric structure. The disulfide bonds are shown in orange. (**B**) Schematic presentations of the changes of HdeB conformational exchanges from neutral to near-neutral pH conditions.

Based on our current results and previous studies, we propose a model for HdeB chaperone function at near-neutral pH: HdeB homo-dimer adopts a ‘spring clamp’ structure in the pH range of 7.0–4.0, with the α2-α3 loops from two subunits resembling the two clamps (Fig. 6). At neutral pH, residues at the dimer interface centering around the ‘clamp’ region show conformational exchanges with k_ex_ ~ 2000 s^−1^, suggesting local dynamics that may allow local structural loosening in the upper part of the dimer. This structural flexibility could prime it for action upon exposure to environmental stress, which may not necessarily be limited to acidic stress. At near-neutral pH (5.0–4.0), HdeB exhibits the highest activity. Residues showing conformational exchanges still cluster around the ‘clamp’ region, but with a slightly increased number of residues involved and a decreased exchange rate (k_ex_ ~ 1000 s^−1^ at pH 4.5/4.0). We suggest that the slower exchange may allow the ‘destabilized’ or ‘activated’ conformation to have a suitable lifetime to interaction with substrate proteins. More residues in helix α2 are involved in conformational exchanges, which may be due to larger motions of the adjacent ‘clamp’, whereas the dimer remains intact and the region around helix α1 is still relatively rigid. At this stage, the highly dynamic behavior of the ‘clamps’ offers plasticity to interact with substrate proteins, either preventing them from stress-induced denaturation/aggregation or helping them to refold into native conformations.

This mechanism is apparently different from that of HdeA, which requires dimer dissociation and partial unfolding to expose the buried hydrophobic surface at low pH^7^,^9^,^10^,^13^,^14^,^15^,^16^. When partially unfolded, the HdeA protein obviously highly dynamic, which concurs with the notion that conformational dynamics plays a central role in its chaperone function. Since HdeA is functionally inactive at pH above 3, we therefore speculate that its conformational dynamics may be significantly different from HdeB. A previous study of HdeA dynamics by solution NMR reveals an overall rigid structure at pH above 4.0^14^. The reported *R*_*2*_*/R*_*1*_ ratios are generally uniform throughout the protein sequence (excluding the flexible N-terminal) at pH 5.0 and 4.0, whereas the conformational heterogeneity slightly increases at pH 3.0, suggesting a loosened tertiary fold^14^. To better compare the dynamics of HdeA/B, we similarly utilized the ^15^N relaxation dispersion experiment to obtain information on HdeA μs-ms timescale conformational exchanges. As anticipated, results at pH 3.0 and 4.0 fail to identify residues showing significant conformational exchanges, strongly supporting the scenario that HdeA is structurally rigid in this pH range. Nevertheless, although HdeA and HdeB exhibit different chaperone activities and adopt distinct mechanisms, protein conformational dynamics plays an important role in both cases.

The HdeA/B proteins may act in relay to protect periplasmic proteins during acid stress as suggested by Dahl *et al.*^17^. When the bacteria enter hosts’ stomach along with food, the environmental pH quickly drops to lower than 3. HdeA undergoes acid-induced activation and protects denatured substrates from aggregating into larger complexes. Although HdeB also dissociates and unfolds at this pH range, its exposed hydrophobic surface is much less compared to HdeA^5^, resulting in a much lower chaperone activity. As the bacteria further move towards the intestine, the environmental pH rises gradually. At near-neutral pH, HdeA refolds and becomes inactivated, whereas HdeB reaches its optimal activity, enabling it to bind substrate proteins and help them refold into native states. Moreover, when bacteria enter hosts’ stomach under fasting conditions (pH ~ 4), the activity of HdeB instead of HdeA would be required for anti-acid response. Taken together, HdeA/B proteins show different pH-dependent dynamic properties and perform complementary physiological functions in protecting periplasmic proteins during acid stress.

The molecular details of how HdeA and HdeB proteins bind substrate proteins are still unclear and present an intriguing topic for deeper understanding of chaperone-substrate interactions. Since chaperone-substrate complexes generally contain large proportion of disordered regions and hence highly dynamic, they are difficult to crystalize and are better suited for investigation by NMR techniques^35^. Recent advances in solution NMR methods offer new probabilities in elucidating high-resolution structural information of chaperone-substrate interactions, as represented by the excellent studies of the bacterial trigger factor chaperone^36^, the ATP-dependent DnaK chaperone^37^,^38^ and the periplasmic membrane protein chaperone Skp^26^. These studies highlight some common features such as the involvement of hydrophobic residues in binding and the highly dynamic properties of the chaperone-substrate interactions, which are consistent with our results.

Moreover, it has been proposed that the disordered structural regions of chaperones may play important functional roles^27^. In the case of ATP-dependent Hsp70 (*E. coli* DnaK) and Hsp60 (*E. coli* GroEL) chaperones, as well as the trigger factor chaperone, the disordered regions appear to act as inter-domain linkers and contribute to conformational rearrangement necessary for substrate interactions^27^,^28^,^38^. Currently available structural data for these chaperones map the substrate binding sites onto surface-exposed hydrophobic areas in structurally ordered domains^27^,^38^, whereas whether the disordered regions directly participate in substrate binding remains unclear. However, a group of ATP-independent chaperones that function during stress conditions, represented by the acid-activated chaperone HdeA and the oxidative stress-activated chaperone Hsp33, undergo stress induced order to disorder transition that activates the chaperone function^10^,^27^,^29^. Both proteins are classified as conditionally disordered chaperones and the stress-induced disordered structural regions are essential for direct binding with substrates. In particular, the study by Reichmann and co-workers implies that Hsp33 and DnaK share overlapping substrates but show distinct folding-state specificities (the disordered segment of Hsp33 preferably binds early unfolding intermediates with residual secondary structures, whereas DnaK favors more extended unstructured polypeptides), and leads to a hypothesis of a hierarchical substrate binding, transfer and refolding mechanism^29^. A similar notion might also hold true in the periplasmic HdeA/B chaperone system in which HdeA requires an order to disorder transition for interacting and disaggregating substrate proteins at low pH, whereas HdeB may utilize its dynamic disordered loop to protect substrate proteins at near-neutral conditions. The distinct conformational and dynamic states of HdeA and HdeB may also result in differential preferences for the folding states of substrates and thus confer to a hierarchical anti-acid chaperoning mechanism. Further investigations on HdeA/B-substrate complexes would provide invaluable insights in the relationship between protein disorder and chaperone function.

## Methods

### Protein Expression and Purification

The pET-28a(+) plasmid (Novagen) harboring the *hdeB* gene was transformed into *E. coli* BL21(DE3)-T1R stain (Sigma-Aldrich) for expression. The culture was first grown in Luria-Bertani medium, then centrifuged and resuspended in M9 medium with antibiotics (kanamycin) and ^15^NH_4_Cl with or without ^13^C_6_-glucose for preparations of ^13^C/^15^N-labeled or ^15^N-labeled samples. Protein overexpression was induced by adding isopropyl-β-D-thiogalactoside (IPTG) to a final concentration of 0.4 mM at OD_600_ = 0.7. After 8 hr induction at 37 °C, the cell was harvested and frozen at -80 °C. The cell pellet was resuspended in a buffer containing 50 mM sodium phosphate, 45 mM citric acid (pH 7.0) and lysed by sonication. The HdeB protein was purified by acid precipitation at pH 3.0 and neutralization back to 7.0. After a subsequent gel-filtration chromatography (Superdex-75) using an ÄKTA FPLC system (GE Healthcare), we were able to obtain protein samples with purity >90% as judged by SDS-PAGE. The NMR samples were prepared in a buffer containing 50 mM sodium phosphate and 45 mM citric acid at pH ranging from 7.0 to 2.0. The sample at pH 1.5 was prepared in 30 mM NaCl and the pH was adjusted by HCl. D_2_O was added to 10% for field lock and 2,2-dimethyl-2-silapentanesulfonic acid was used as the internal chemical shift reference.

### NMR Spectroscopy

The NMR experiments were carried out at 25 °C on Bruker Avance 500-, 700- and 800-MHz spectrometers equipped with four RF channels and triple resonance probes with pulsed field gradients. The chemical shift assignments were obtained by conventional triple resonance experiments^39^ using ^13^C/^15^N-labeled HdeB sample at pH 4.5 and 1.5. Three-dimensional (3D) ^15^N- and ^13^C-edited NOESY-heteronuclear single quantum coherence (HSQC) spectra (mixing times 100 ms) for HdeB at pH 4.5 and 1.5 were collected to confirm the assignments and obtain distance restraints. 3D ^13^C-edited NOESY-HSQC spectrum for aromatic residues (mixing time 100 ms) was collected to assign aromatic residues and obtain distance restraints. 3D ^13^C/^15^N-filtered ^13^C-edited NOESY-HSQC experiments (mixing times 100 and 200 ms) were recorded using a ^13^C/^15^N-labeled and unlabeled mixed HdeB sample to obtain inter-subunit distance restraints. All NMR spectra were processed using NMRPipe^40^ and analyzed using NMRView^41^.

### Structure Calculations

The structure of dimeric HdeB was calculated at pH 4.5. Distance restraints were derived from inter-proton NOEs. Inter-subunit distance restraints were extracted from the ^15^N- and ^13^C-edited NOESY-HSQC spectra using the X-ray crystal structure of HdeB (protein data bank accession number 2XUV) as a reference, and further confirmed by the ^13^C/^15^N-filtered ^13^C-edited NOESY-HSQC experiments. Dihedral angles (Φ and ψ) were determined from backbone chemical shifts using the program TALOS^42^. The initial structures were calculated with the CANDID module of the CYANA program^43^,^44^. The 20 lowest energy structures were selected as models for SANE to extend the NOE assignments^45^. Two hundred structures were calculated by CYANA, and the 100 lowest energy structures were used as initial structures and refined using AMBER^46^. Finally, the 20 lowest-energy conformers were selected as representative structures.

### Backbone ^15^N Relaxation Measurements

The backbone ^15^N relaxation parameters, including the longitudinal relaxation rates (*R*_*1*_), transverse relaxation rates (*R*_*2*_), and steady-state heteronuclear {^1^H}-^15^N NOE values of HdeB were measured at pH 7.0, 5.0, 4.5, 4.0, 3.2, 2.8, 2.5, 2.0 and 1.5^47^. All data were collected at 25 °C on a Bruker Avance 800-MHz spectrometer. The relaxation rate constants were obtained by fitting the peak intensities to a single exponential function using the nonlinear least squares method as described^48^. The {^1^H}-^15^N NOE experiments were recorded in the presence and absence of a 3-s proton pre-saturation period prior to the ^15^N excitation pulse and using recycle delays of 2 and 5 s, respectively. The data were analyzed using the Lipari-Szabo model-free formalism to extract the microdynamic parameters^49^,^50^,^51^. Briefly, the ^15^N relaxation data were interpreted in terms of motion of the N-H bond by fitting with five models with increasing complexity. The models include model M1 (*S*^*2*^), model M2 (*S*^*2*^, *τ*_*e*_), model M3 (*S*^*2*^, *R*_*ex*_), model M4 (*S*^*2*^, *τ*_*e*_, *R*_*ex*_), and model M5 (*S*^*2*^, *τ*_*e*_, *S*^*2*^_*f*_), where *S*^*2*^ is the squared order parameter, *τ*_*e*_ is a correlation time describing the internal motion on ps-ns time scales, *S*^*2*^_*f*_ is a second squared order parameter describing the fast internal motion on ps-ns time scales, and *R*_*ex*_ is the chemical exchange contribution to *R*_*2*_. Standard errors of the microdynamic parameters were obtained by Monte Carlo simulations.

### Backbone ^15^N Relaxation Dispersion Measurements

^15^N Carr-Purcell-Meiboom-Gill (CPMG) relaxation dispersion experiments were acquired at 25 °C on Bruker Avance 600- and 800-MHz spectrometers for HdeB samples at pH 6.0, 4.5 and 4.0^30^,^31^. A constant transverse relaxation time of 60 ms was used for all samples. Data were recorded for fourteen different ν_CPMG_ values of 0, 50, 100(x2), 150, 200, 250, 300, 350, 400, 450, 500, 600 and 750 Hz at two static fields with ^1^H frequency of 800.2 MHz and 600.13 MHz. Here ν_CPMG_ = 1/(4τ_cp_), where τ_cp_ is the time between refocusing pulses during the CPMG pulse train. Peak intensities were measured using NMRView^41^ and the effected transverse relaxation rates *R*_*2*_^*eff*^ were determined using the equation *R*_*2*_^*eff*^ = (-1/τ_relax_)ln(*I*_*νCPMG*_/*I*_*0*_), where τ_relax_ is the constant transverse relaxation time, *I*_*0*_ is the intensity measured in the reference spectrum, and *I*_*νCPMG*_ is the intensity measured at different CPMG field strengths ν_CPMG_. Residues exhibiting conformational exchanges on appropriate timescales would show a dispersion profile of *R*_*2*_^*eff*^ values dependent on ν_CPMG_. All dispersion data were fitted with a two-state exchange model using the general expression without assumption regarding the exchange regime^32^,^33^. To extract the exchange rate constant k_ex_ between states A and B, the populations p_A_ and p_B_, and the ^15^N chemical shift differences Δω between states A and B for each residue, global fitting of data obtained at pH 6.0, 4.5 and 4.0 were performed using a program from L. Kay and D. Korzhnev with a numerical approximation of the Bloch-McConnell equation^33^,^34^. The dispersion curves for individual residues were generated using the GLOVE program^52^ by fixing the k_ex_, p_A_p_B_ and Δω parameters.

## Additional Information

**Accession codes**: The atomic coordinates of HdeB solution structure have been deposited in the Protein Data Bank under the accession code 2MYJ.

**How to cite this article**: Ding, J. *et al.* HdeB chaperone activity is coupled to its intrinsic dynamic properties. *Sci. Rep.* **5**, 16856; doi: 10.1038/srep16856 (2015).

## Supplementary Material

Supplementary Information

## Figures and Tables

**Table 1:** Structural statistics of *E. coli* HdeB.

Distance restraints	
Total unambiguous NOEs^a^	2515
Intra-residue	1035
Sequential (|i-j| = 1)	623
Medium-range (1 <|i-j| < 5)	434
Long-range (|i–j| >= 5)	292
Total Ambiguous NOEs	799
Intermolecular NOEs^b^	131
Dihedral angle restraints	
φ	76
ψ	76
Number of restraint violations	
Distance violations (>0.3 Å)^c^	0
Dihedral angle violations (>10°)^d^	0
Deviations from ideal geometry	
Covalent bond lengths (Å)	0.015 ± 0.001
Covalent angles (°)	2.34 ± 0.04
RMSD from mean structure (Å)	
Secondary-structure backbone atoms	0.7 ± 0.1
Secondary-structure heavy atoms	1.0 ± 0.2
All backbone atoms	1.0 ± 0.2
All heavy atoms	1.4 ± 0.4
Ramachandran statistics (%)	
Most favored regions	83.8
Additional allowed regions	12.9
Generously allowed regions	1.3
Disallowed regions	2.0
Energy (kcal/mol)	
Mean AMBER energy	-5040.3 ± 13.2
NOE restraint violation energy	52.2 ± 3.8
Angle restraint violation energy	2.9 ± 0.5

^a^The number of distance restraints is calculated for a single monomer.

^b^The number of intermolecular distance restraints is calculated for a single monomer, whereas during structure calculation the restraints were duplicated to account for symmetry.

^c^The largest distance violation is 0.23 Å.

^d^The largest angle violation is 6.73°.
