# Supplementary material for: HdeB chaperone activity is coupled to its intrinsic dynamic properties
Source: Sci Rep. 2015 Nov 23;5:16856. doi: 10.1038/srep16856 (PMC4655364; doi:10.1038/srep16856)
Supplement: Supplementary Information [file srep16856-s1.pdf]

Supplementary Information for "HdeB chaperone activity is coupled to its intrinsic dynamic properties"

Jienv Ding, Chengfeng Yang, Xiaogang Niu, Yunfei Hu*, Changwen Jin*

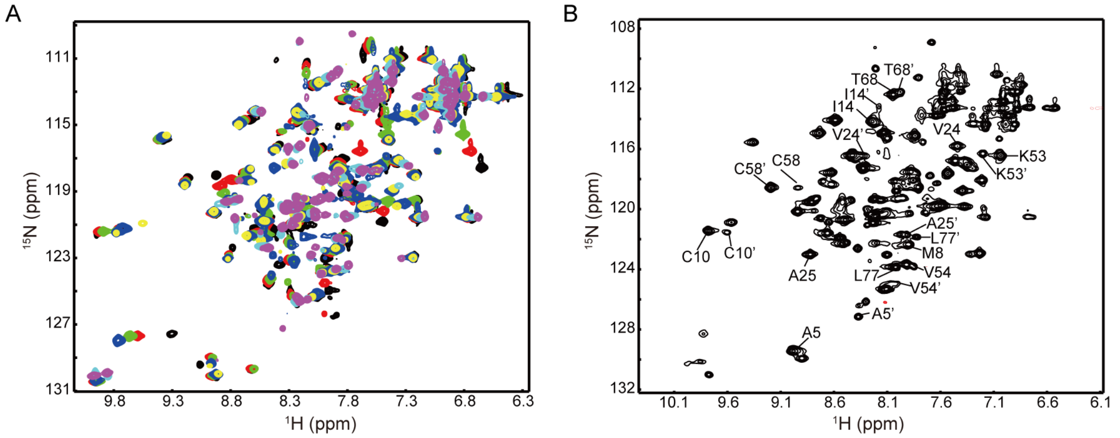

Fig. S1. pH titration of *E. coli* HdeB monitored by 2D NMR. (A) Overlay of 2D ^{1}H-^{15}N HSQC spectra of HdeB at pH 7.0 (black), 4.5 (red), 4.0 (green), 3.2 (blue), 2.8 (yellow), 2.0 (cyan) and 1.5 (magenta). (B) 2D ^{1}H-^{15}N HSQC spectrum of HdeB at pH 2.8 showing multiple conformational states with representative residues labeled.

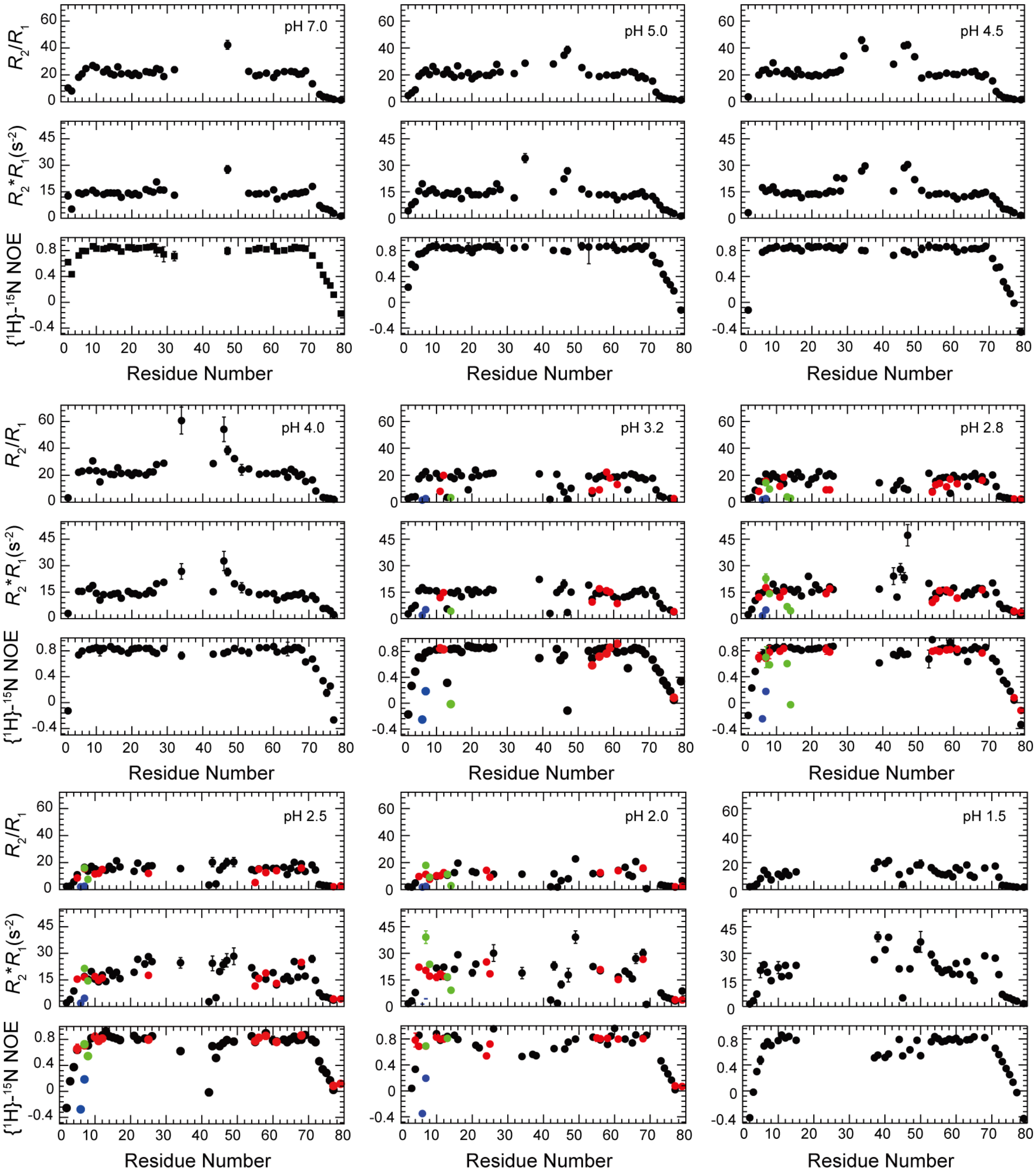

Fig. S2. Backbone ^{15}N relaxation parameters R_2/R_1, R_2*R_1 and {^{1}H}-^{15}N NOE values of *E. coli* HdeB at different pH conditions. Residues with multiple conformational states at pH 3.2-2.0 are presented with different colors.

Fig. S3-a

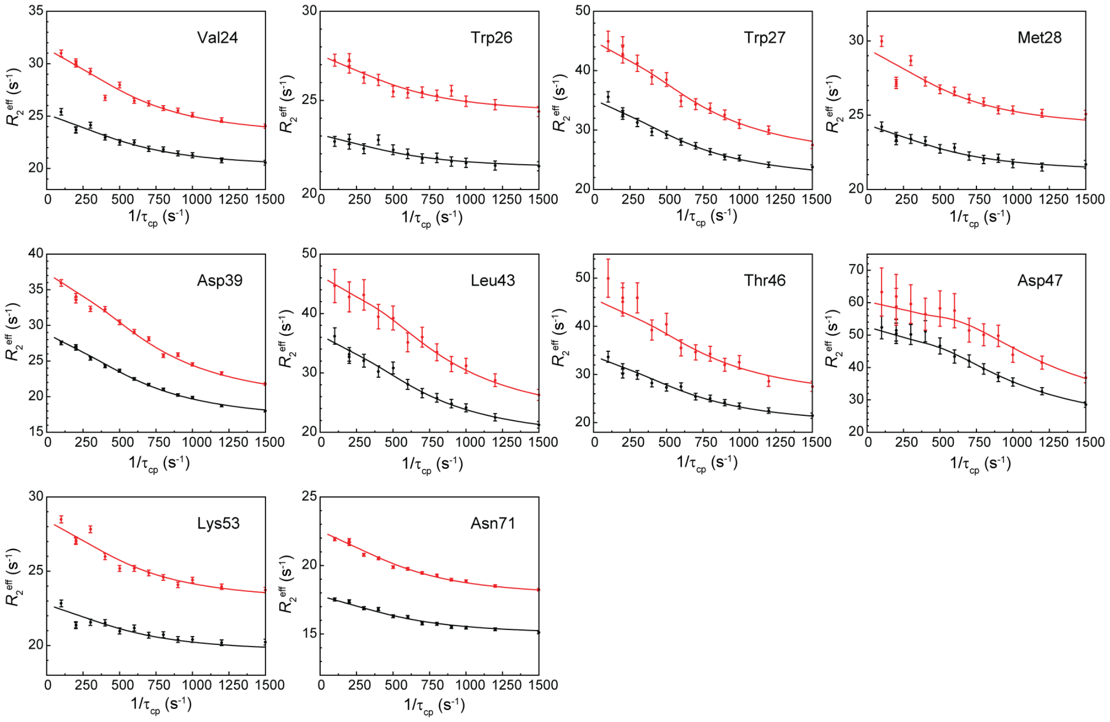

Fig. S3-a. Relaxation dispersion curves of *E. coli* HdeB at pH 6.0. Data measured at ^{1}H frequency of 600.13 and 800.2 MHz static fields are shown in black and red, respectively. The data were fitted globally.

Fig. S3-b

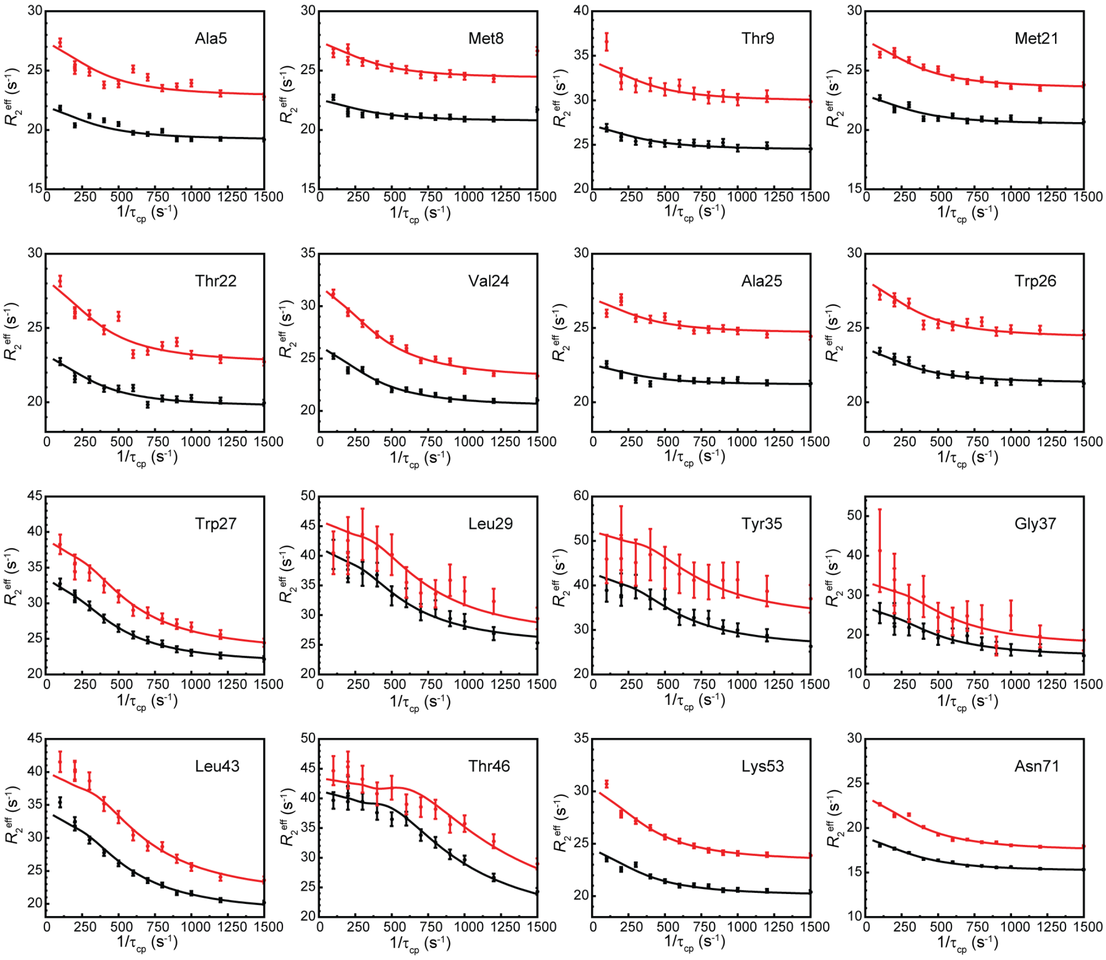

Fig. S3-b. Relaxation dispersion curves of *E. coli* HdeB at pH 4.5. Data measured at ^{1}H frequency of 600.13 and 800.2 MHz static fields are shown in black and red, respectively. The data were fitted globally.

Fig. S3-c

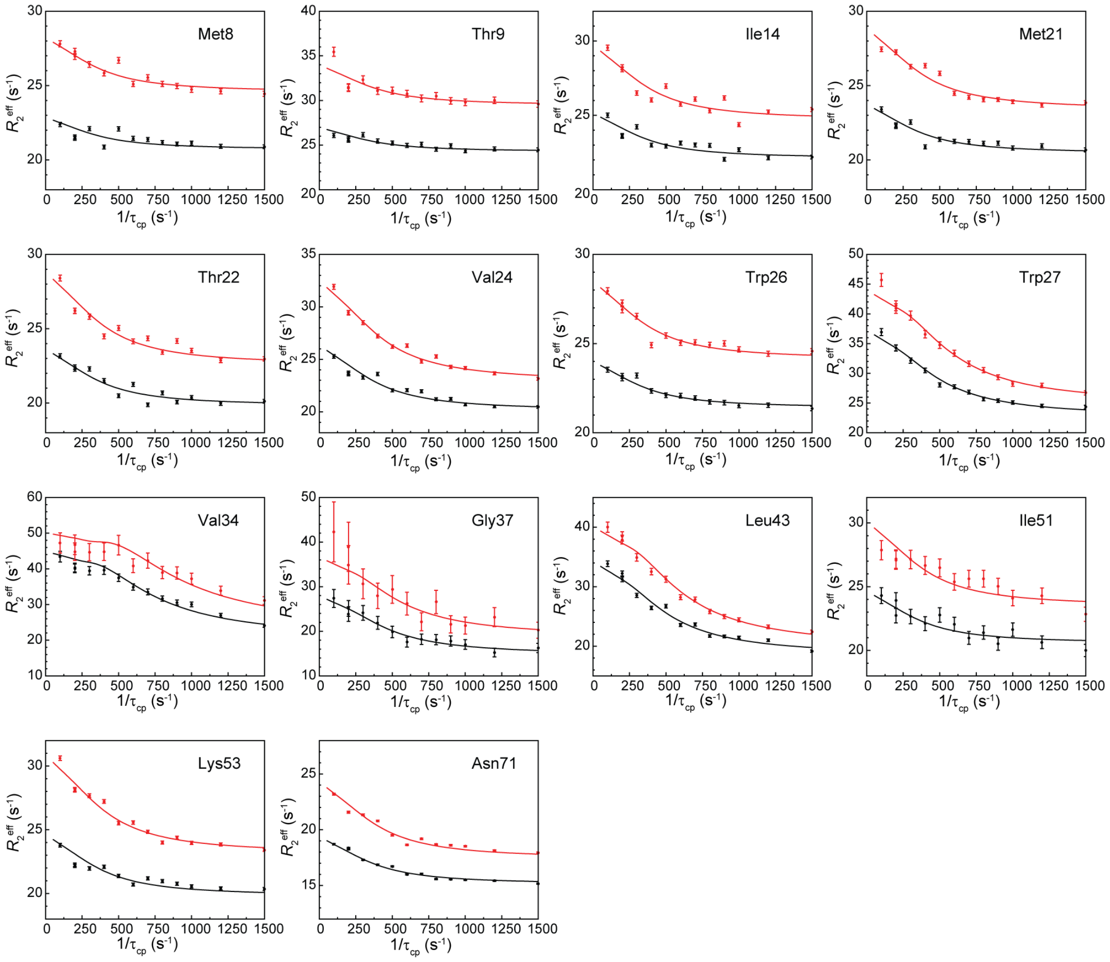

Fig. S3-c. Relaxation dispersion curves of *E. coli* HdeB at pH 4.0. Data measured at ^{1}H frequency of 600.13 and 800.2 MHz static fields are shown in black and red, respectively. The data were fitted globally.

Table S1: **Summary of the ^{15}N CPMG relaxation dispersion data analysis results of HdeB at pH 6.0, 4.5 and 4.0 using global fitting.**

pH 6.0 (global fitting)		pH 4.5 (global fitting)		pH 4.0 (global fitting)	
k_{ex}	1822 ± 62	k_{ex}	1095 ± 37	k_{ex}	1095 ± 44
p_b	0.030 ± 0.002	p_b	0.020 ± 0.001	p_b	0.029 ± 0.002
Residue	$\|\Delta\omega\|$ (ppm)	Residue	$\|\Delta\omega\|$ (ppm)	Residue	$\|\Delta\omega\|$ (ppm)
24	1.64 ± 0.06	5	0.99 ± 0.04	8	0.78 ± 0.05
26	0.97 ± 0.06	8	0.79 ± 0.05	9	0.88 ± 0.09
27	2.97 ± 0.15	9	0.98 ± 0.09	14	0.94 ± 0.05
28	1.27 ± 0.06	21	0.91 ± 0.04	21	0.98 ± 0.05
39	2.77 ± 0.11	22	1.12 ± 0.05	22	1.07 ± 0.05
43	3.57 ± 0.21	24	1.51 ± 0.05	24	1.41 ± 0.07
46	3.08 ± 0.18	25	0.67 ± 0.04	26	0.87 ± 0.05
47	6.36 ± 0.64	26	0.89 ± 0.05	27	2.60 ± 0.17
53	1.28 ± 0.05	27	2.56 ± 0.12	34	5.06 ± 0.56
71	1.20 ± 0.04	29	3.60 ± 0.39	37	2.39 ± 0.43
		35	3.70 ± 0.50	43	2.80 ± 0.17
		37	2.67 ± 0.37	51	1.11 ± 0.15
		43	3.32 ± 0.14	53	1.22 ± 0.05
		46	6.79 ± 0.40	71	1.13 ± 0.04
		53	1.29 ± 0.04		
		71	1.17 ± 0.03		
